# SGLT2 inhibitors enhance ketogenesis by acting as allosteric activators of the mitochondrial enzyme HMGCS2

**DOI:** 10.1172/JCI192333

**Published:** 2026-07-01

**Authors:** Abdualrahman Mohammed Abdualkader, Xiaobei Li, Yiming Yin, Chenhao Bai, Parisa Pourfarziani, Jiaheng Guan, Sora Kwon, Kyoung-Han Kim, Rami Al Batran

**Affiliations:** 1 Faculty of Pharmacy, Université de Montréal, Montréal, Quebec, Canada.; 2 University of Ottawa Heart Institute, Ottawa, Ontario, Canada.

**Keywords:** Endocrinology, Metabolism, Diabetes, Fatty acid oxidation, Glucose metabolism

## Abstract

SGLT2 inhibitors boost ketone production by directly activating a liver enzyme, revealing a new mechanism that may contribute to their heart and kidney benefits

**To the Editor:** The discovery of sodium-glucose cotransporter-2 inhibitors (SGLT2i) has transformed the management of type 2 diabetes, cardiovascular disease, and kidney disease. SGLT2i reduce cardiovascular death, heart failure hospitalizations, and kidney failure progression in patients with or without diabetes (1), indicating benefits beyond glucose lowering. Emerging evidence suggests these cardiorenal benefits are partly mediated by increases in plasma ketone bodies (2). Although the mechanism of SGLT2i-induced ketosis remains unclear, it is thought to result primarily from glycosuria-driven hepatic ketogenesis. Notably, this ketosis occurs independent of diabetes status, suggesting additional off-target mechanisms beyond glycemic control.

To test this, we acutely treated normoglycemic, 6-hour-fasted C57BL/6J male mice with the SGLT2i empagliflozin (EMPA) and assessed ketosis 5 minutes later, before any detectable changes in glycemic parameters. EMPA rapidly increased circulating β-hydroxybutyrate (βOHB) levels and enhanced hepatic 3-hydroxy-3-methylglutaryl-CoA synthase 2 (HMGCS2) activity (Figure 1, A and B) dose-dependently (Supplemental Figure 1, A and B; supplemental material available online with this article; https://doi.org/10.1172/JCI192333DS1). These effects occurred without changes in glucose, glycosuria, non-esterified fatty acids (NEFA), glycerol, insulin/glucagon ratio, or expression of lipolysis and fatty acid oxidation genes (Figure 1, C–G, and Supplemental Figure 1, C and D). EMPA did not increase βOHB or HMGCS2 activity under fed conditions, nor did it alter these metabolic parameters (Supplemental Figure 2, A–H), likely reflecting substrate limitation in the fed state. Similarly, EMPA increased βOHB and HMGCS2 activity in 6-hour-fasted Alb^Cre^ control mice but not in liver-specific HMGCS2-deficient mice (*Hmgcs2*^Liver–/–^) (Figure 1, H and I), confirming that EMPA-induced ketogenesis requires hepatic HMGCS2. Importantly, *Hmgcs2*^Liver–/–^ mice displayed normal metabolic parameters on chow diet (Supplemental Figure 3, A–R), suggesting hepatic HMGCS2 deletion does not introduce baseline metabolic alterations that could confound interpretation of acute EMPA responses.

To determine whether EMPA directly binds HMGCS2, we performed drug affinity responsive target stability (DARTs) assay. EMPA reduced protease susceptibility of HMGCS2 in mouse liver lysates without affecting other ketogenic enzymes or HSP90 (Figure 1J), supporting direct EMPA–HMGCS2 interaction. To test whether this effect extends to humans, primary human hepatocytes treated with EMPA showed increased HMGCS2 activity (Figure 1K), also observed within 5 minutes of exposure in HEK293 cells (Supplemental Figure 3S). Consistently, dose-response assays using purified human HMGCS2 revealed that 3 SGLT2i increased enzyme activity (Figure 1L), despite distinct chemical structures and pharmacokinetic profiles. Enzyme kinetic analysis showed that SGLT2i increased both maximum reaction velocity (*V_max_*) and Michaelis-Menten constant (*K_m_*) (Figure 1M). Lineweaver-Burk analysis indicated that this pattern reflects increased catalytic turnover with reduced substrate affinity (Figure 1N), consistent with allosteric activation of HMGCS2 rather than substrate competition.

To identify binding sites between EMPA and HMGCS2 and determine whether these interactions are conserved across different SGLT2i, we conducted molecular docking studies and found that all tested SGLT2i bind favorably near the catalytic domain of HMGCS2 (Supplemental Figure 4A). For example, EMPA is predicted to form a hydrogen bond with His301, a residue within the Cys166–His301–Glu132 catalytic triad (Figure 1O). Molecular dynamics simulations further showed that EMPA binding induces local conformational changes that shorten the distance between His301 and the catalytic nucleophile Cys166 (Figure 1P). This rearrangement stabilizes interactions within the catalytic triad and promotes a catalytic configuration positioning Cys166 for nucleophilic attack on acetyl-CoA, facilitating initiation of the enzymatic reaction (3). Because EMPA does not occupy the acetyl-CoA substrate-binding tunnel, these findings support a mechanism in which SGLT2i enhance HMGCS2 activity through allosteric modulation of the catalytic domain rather than substrate competition.

To explore whether SGLT2i binding influences posttranslational modifications of HMGCS2, we performed mass spectrometry on HMGCS2 immunoprecipitated from mouse livers treated with vehicle or EMPA. MS analysis revealed that EMPA treatment was associated with reduced phosphorylation of Thr91, a residue near the entrance of the HMGCS2 active-site tunnel, without altering global hepatic phosphatase activity (Figure 1, Q and R). To determine whether this effect resulted from phosphatase activation or conformational modulation, mice were treated with the broad phosphatase inhibitor okadaic acid (OA) ± EMPA. Although Thr91 phosphorylation was undetectable in liver samples treated with OA regardless of EMPA exposure (data not shown), OA increased HMGCS2 activity and EMPA further enhanced this effect (Figure 1S), indicating EMPA does not activate OA-sensitive phosphatases. To assess the functional relevance of Thr91 phosphorylation, we generated phospho-null (T91A) and phosphomimetic (T91D) HMGCS2 mutants. Functional assays showed that Thr91 phosphorylation inhibits HMGCS2 activity, as T91D reduced activity whereas T91A increased it. Notably, EMPA increased activity in T91A but failed to rescue activity in T91D (Figure 1T and Supplemental Figure 4, B–H). Together, these findings suggest EMPA stabilizes a catalytically favorable conformation of HMGCS2 compatible with the dephosphorylated state of Thr91, consistent with an allosteric mechanism of enzyme activation.

In conclusion, these findings identify HMGCS2 as a direct target of SGLT2i and reveal that SGLT2i promote ketogenesis via allosteric activation of HMGCS2. The physiological relevance of this mechanism in humans remains to be determined. Finally, while SGLT2 deficiency promotes fasting ketonemia through glycosuria (4), our findings suggest an additional mechanism whereby SGLT2i rapidly enhance HMGCS2 activity.

## Conflict of interest

The authors have declared that no conflict of interest exists.

## Funding support

Project Grant (PJT-195730) from the Canadian Institutes of Health Research (CIHR) to RA.

## Supplementary Material

Supplemental data

Unedited blot and gel images

Supporting data values

## Figures and Tables

**Figure 1:**
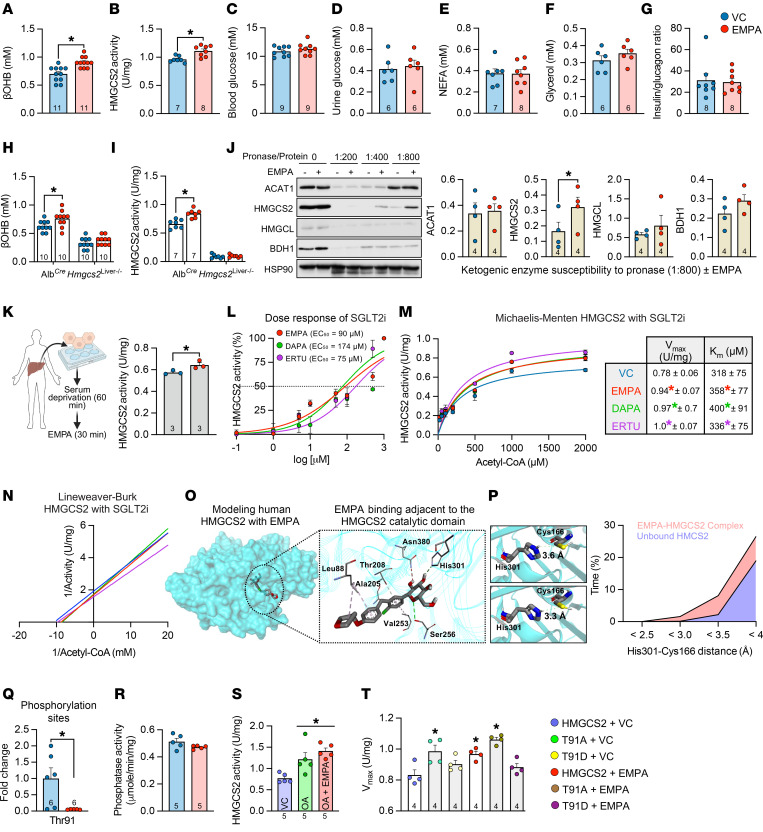
SGLT2i as allosteric activators of HMGCS2. (**A**–**G**) βOHB levels, HMGCS2 activity, and metabolic parameters in C57BL/6J mice fasted for 6 hours and treated with vehicle (VC) or empagliflozin (EMPA; 30 mg/kg). (**H** and **I**) βOHB levels and HMGCS2 activity in 6-hour-fasted Alb*^Cre^* and *Hmgcs2*^Liver–/–^ mice treated with VC or EMPA. (**J**) DARTs assay of ketogenic enzymes in liver lysates ± EMPA (3 mM). HSP90 served as loading control. (**K**) HMGCS2 activity in human hepatocytes treated with VC or EMPA (100 μM). (**L**) Dose–response activation of recombinant human HMGCS2 by 3 SGLT2i (EMPA, dapagliflozin [DAPA], ertugliflozin [ERTU]); EC_50_ values indicated. (**M** and **N**) Kinetic analyses of HMGCS2 with SGLT2i; *V_max_* and *K_m_* values are shown to the right and expressed as mean ± SEM. (**O**) Molecular docking of EMPA highlighting interacting residues. (**P**) Molecular dynamics simulations showing reduced His301–Cys166 distance upon EMPA binding; top, unbound; bottom, EMPA-bound. (**Q**) Phosphorylation sites identified in HMGCS2 immunoprecipitates from fasted mice treated with VC or EMPA. (**R**) Global hepatic phosphatase activity following VC or EMPA treatment. (**S**) HMGCS2 activity in mice treated with OA (150 μg/kg) ± EMPA. (**T**) *V_max_* of purified human WT, T91A, and T91D HMGCS2 mutants ± EMPA. Data are mean ± SEM. Statistical significance was determined using 2-tailed Student’s *t* test or 1-way ANOVA. *P < 0.05 vs. VC.

## Version 1 07/01/2026

Electronic publication
