# Supplementary material for: SGLT2 inhibitors enhance ketogenesis by acting as allosteric activators of the mitochondrial enzyme HMGCS2
Source: J Clin Invest. 2026 Jul 1;136(13):e192333. doi: 10.1172/JCI192333 (PMC13318103; doi:10.1172/JCI192333)
Supplement: Supplemental data [file jci-136-192333-s233.pdf]

SUPPLEMENTAL MATERIAL For

SGLT2 inhibitors enhance ketogenesis by acting as allosteric activators of the mitochondrial enzyme HMGCS2

Abdualrahman Mohammed Abdualkader[1], Xiaobei Li[1], Yiming Yin[1], Chenhao Bai[1], Parisa Pourfarziani[1], Jiaheng Guan[1], Sora Kwon[2], Kyoung-Han Kim[2], Rami Al Batran[1*]

[1]Faculty of Pharmacy, Université de Montréal, Montreal, QC, Canada
[2]University of Ottawa Heart Institute, Ottawa, ON, Canada

Correspondence:
Dr. Rami Al Batran
Faculty of Pharmacy, Université de Montréal
2940 Chemin de la Polytechnique
Montréal, QC H3T 1J4, Canada
Tel: +1-514-343-6111 ext. 14500
Email: rami.al.batran@umontreal.ca

Contents:

Supplemental Figures 1-4
Methods
References

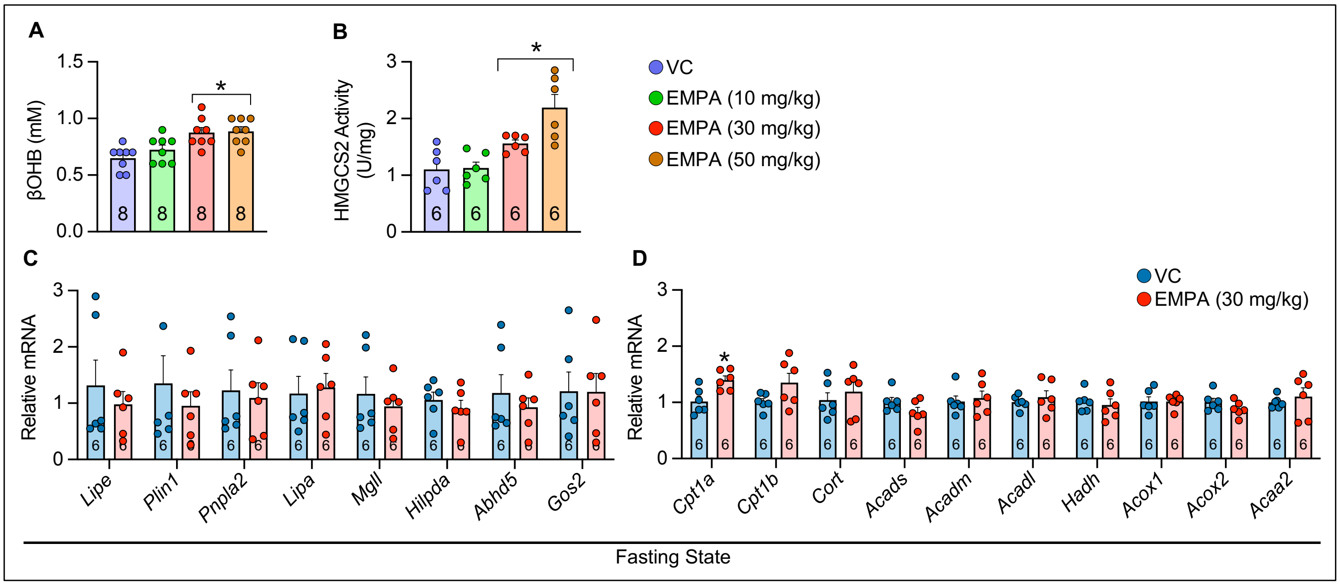

Supplemental Figure 1: The SGLT2 inhibitor empagliflozin (EMPA) induces ketosis in a dose-dependent manner independent of metabolic shifts toward lipolysis and fatty acid oxidation. (**A**) Blood levels of β-hydroxybutyrate (βOHB) and (**B**) hepatic HMGCS2 activity in C57BL/6J mice fasted for 6 hours and treated orally with either vehicle control (VC) or empagliflozin (EMPA, 10, 30, 50 mg/kg). (**C, D**) Relative mRNA expression of genes related to lipolysis (**C**) and hepatic fatty acid oxidation (**D**) after treatment with VC or EMPA (30 mg/kg). Data are presented as means ± SEM with individual data points, and statistical analyses were performed as described in the Statistical Analysis section.

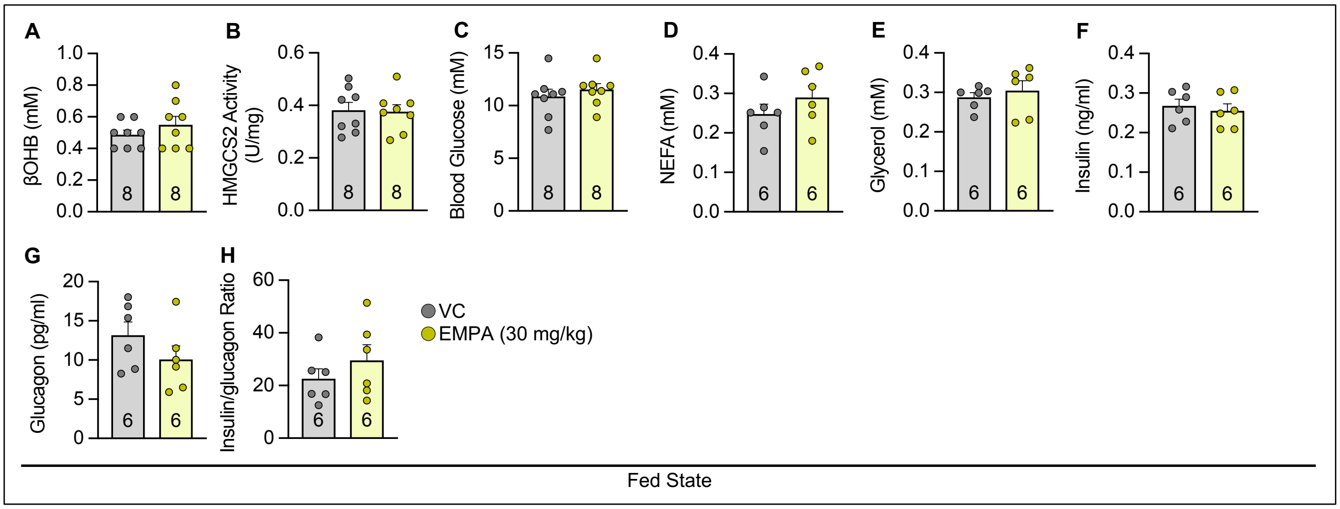

Supplemental Figure 2: The SGLT2 inhibitor empagliflozin (EMPA) fails to induce ketosis in the fed state.

(**A**) Blood levels of β-hydroxybutyrate (βOHB), (**B**) hepatic HMGCS2 activity, and (**C-H**) measurements of blood glucose, non-esterified fatty acids (NEFA), free glycerol, insulin, glucagon, and the insulin-to-glucagon ratio in fed C57BL/6J mice treated orally with either vehicle control (VC) or empagliflozin (EMPA, 30 mg/kg). Data are presented as means ± SEM with individual data points, and statistical analyses were performed as described in the Statistical Analysis section.

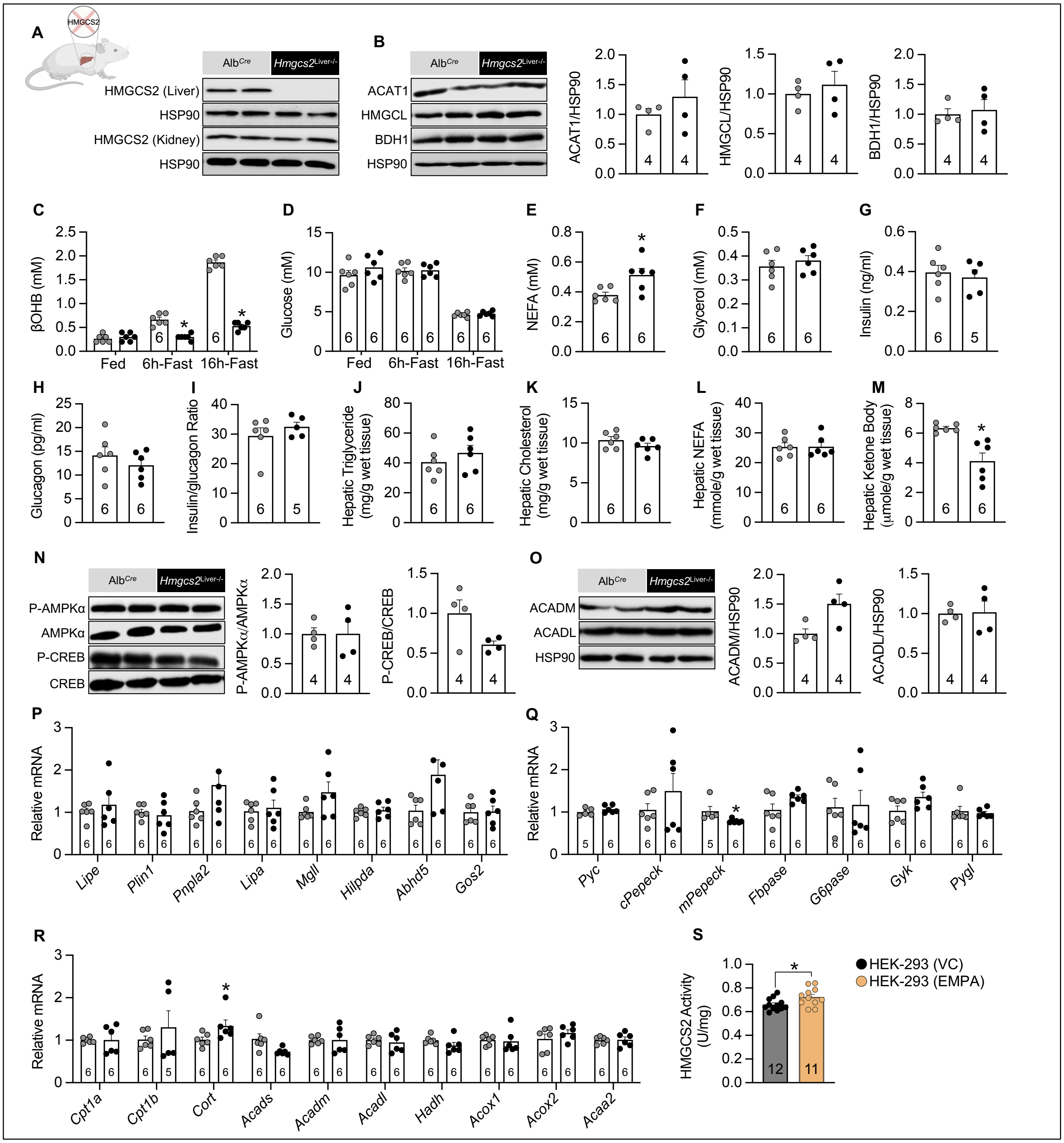

Supplemental Figure 3: Phenotypic characterization of Alb^{Cre} and *Hmgcs2*$^{liver-/-}$ mice under short-term fasting and validation of empagliflozin (EMPA)-induced HMGCS2 activation in HEK-293 cells.

(**A**) Representative immunoblots of HMGCS2 and HSP90 demonstrate the specific deletion of HMGCS2 in the liver. (**B**) Representative immunoblots and quantification of ketogenic enzymes in liver lysates from 6-hour-fasted Alb^{Cre} and $\textit{Hmgcs2}^{\text{liver-/-}}$ mice. (**C, D**) Blood β-hydroxybutyrate (βOHB) and glucose levels in Alb^{Cre} and $\textit{Hmgcs2}^{\text{liver-/-}}$ mice under fed, 6-hour-fasted, and 16-hour-fasted conditions. (**E-I**) Blood glucose, non-esterified fatty acids (NEFA), free glycerol, insulin, glucagon, and the insulin-to-glucagon ratio in 6-hour-fasted Alb^{Cre} and $\textit{Hmgcs2}^{\text{liver-/-}}$ mice. (**J-M**) Hepatic contents of triglycerides, cholesterol, NEFA, and total ketones in 6-hour-fasted Alb^{Cre} and $\textit{Hmgcs2}^{\text{liver-/-}}$ mice. (**N**) Representative immunoblots and quantification of glucagon downstream targets in the liver of 6-hour-fasted Alb^{Cre} and $\textit{Hmgcs2}^{\text{liver-/-}}$ mice. (**O**) Representative immunoblots and quantification of β-oxidation enzymes in the liver of 6-hour-fasted Alb^{Cre} and $\textit{Hmgcs2}^{\text{liver-/-}}$ mice. (**P-R**) mRNA expression of genes related to lipolysis, hepatic gluconeogenesis, and fatty acid oxidation in 6-hour-fasted Alb^{Cre} and $\textit{Hmgcs2}^{\text{liver-/-}}$ mice. (**S**) HMGCS2 activity in human embryonic kidney (HEK-293) cells serum-starved for 60 minutes and subsequently treated with vehicle control (VC) or empagliflozin (EMPA, 100 μM) for 5 minutes. Data are presented as means ± SEM with individual data points, and statistical analyses were performed as described in the Statistical Analysis section.

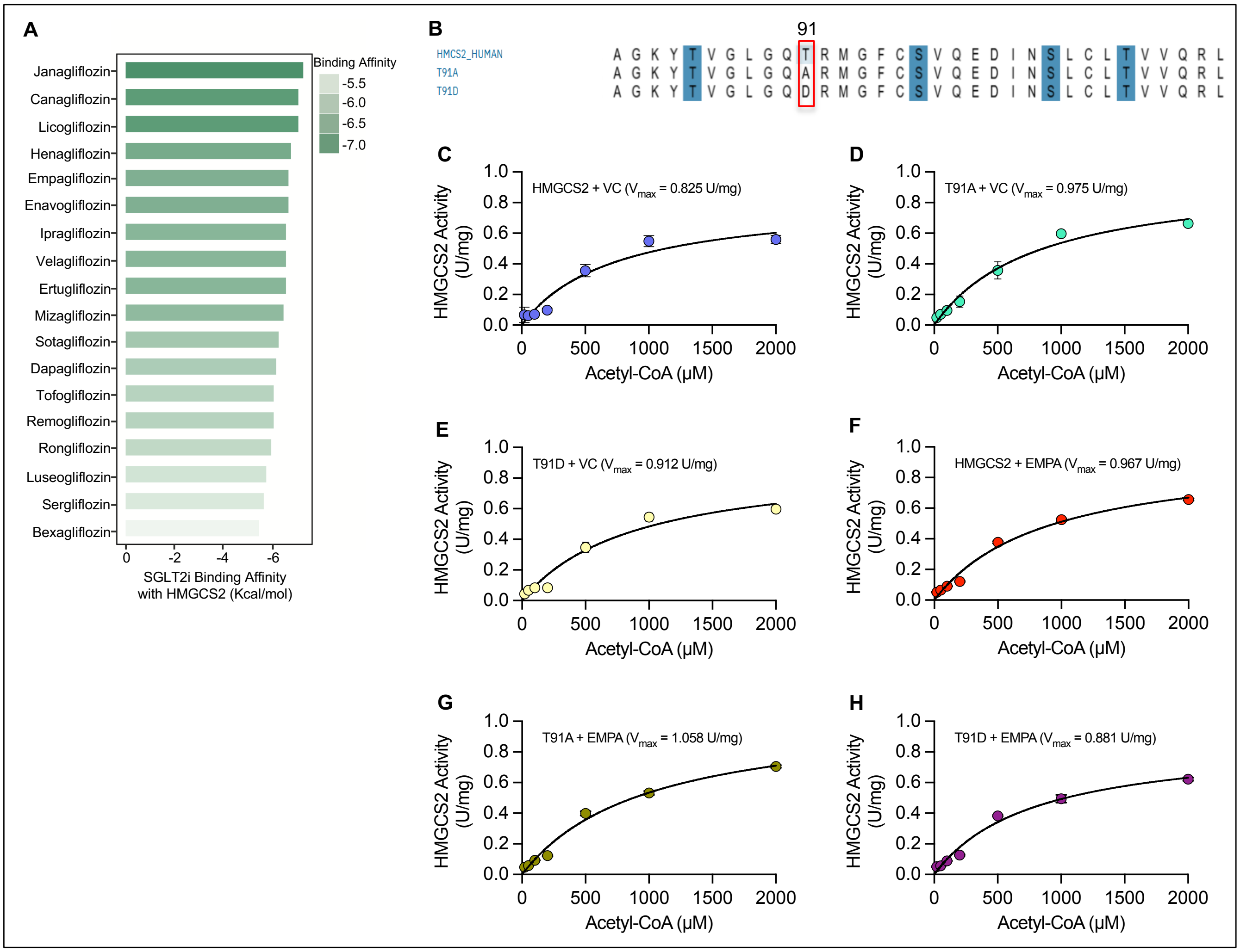

Supplemental Figure 4: Molecular docking and functional validation of SGLT2 inhibitor-mediated regulation of HMGCS2 activity.

(**A**) The docking score illustrates the predicted binding affinity between various SGLT2i and HMGCS2. (**B**) Representation of wild-type human HMGCS2, phospho-null (T91A), and phosphomimetic (T91D) HMGCS2 mutants. (**C-H**) Kinetic analysis of wild-type human HMGCS2, T91A and T91D activity treated with either vehicle control (VC) or 100 μM EMPA. Data are presented as means ± SEM with individual data points, and statistical analyses were performed as described in the Statistical Analysis section.

Materials and Methods

Sex as a biological variable

This study was conducted exclusively in male mice. The potential influence of sex was not assessed and represents a limitation of the current study. Future investigations will be required to determine whether these findings extend to female mice.

Mouse studies

C57BL/6J mice (Strain #000664) were obtained from Jackson Laboratory and bred in-house at the Institute for Research in Immunology and Cancer (IRIC). Liver-specific *Hmgcs2* knockout mice (*Hmgcs2*$^{\text{Liver-/-}}$) were generated by crossing homozygous *Hmgcs2* mice (Strain #068091-JAX) with Albumin-Cre mice (Alb^{Cre}, Strain #003574). All mice were housed in a specific-pathogen-free facility under a 12-hour light/dark cycle at 22°C with 40-55% humidity and maintained on a standard chow diet (Teklad Global Rodent Diets®, 2918). Mice were housed in groups of up to four per cage with appropriate bedding, and had *ad libitum* access to food and water. For in vivo sodium-glucose cotransporter-2 (SGLT2) inhibitor treatment, 7-8-week-old C57BL/6J, *Hmgcs2*$^{\text{Liver-/-}}$, or Alb^{Cre} mice were fasted for 6 hours before receiving an acute oral treatment with either vehicle control (5% DMSO, 40% polyethylene glycol PEG-300, 5% tween-80, and 50% saline) or the SGLT2 inhibitor empagliflozin (EMPA; MedChemExpress, Cat# HY-15409) at a dose of 30 mg/kg. For dose-response experiments, 6-hour-fasted C57BL/6J mice were treated orally with EMPA at 10, 30, or 50 mg/kg. For phosphatase inhibition experiments, 6-hour-fasted C57BL/6J mice received an intraperitoneal injection of okadaic acid (OA; SignalChem Biotech, Cat# P16902100) at 150 µg/kg, followed 10 minutes later by oral administration of either vehicle or EMPA (30 mg/kg). All mice were euthanized 5 minutes after EMPA treatment by intraperitoneal injection of sodium pentobarbital (100 mg/kg) and 2% lidocaine. Tissue samples were collected, immediately frozen, stored at -80°C, and thawed only once prior to analysis.

Metabolite and hormone measurements

Blood glucose and β-hydroxybutyrate (βOHB) levels were measured from tail blood using the Contour® Next One Blood Glucose Meter (Bayer) and the Freestyle Precision Neo Blood Ketone Monitoring System (Abbott), respectively. Urinary glucose concentrations were determined using a colorimetric assay kit (Abcam, Cat# AB65333). Blood samples were collected via cardiac puncture into EDTA-treated, non-heparinized tubes and immediately centrifuged to isolate plasma, as previously described (1). Plasma insulin (ALPCO Diagnostics, Cat# 80-INSHU-E01.1), glucagon (Crystal Chem, Cat# 81518), non-esterified fatty acids (NEFA; Fujifilm, Cat# 999-34691, 995-34791, 991-34891, 993-35191 and 276-76491), and free glycerol (Abcam, Cat# AB5337) concentrations were quantified using ELISA kits.

Tissue triglyceride (Fujifilm, Cat# 994-02891, 990-02991, 464-01601 and 416-00102), cholesterol (Fujifilm, Cat# 999-02601 and 416-00102), and total ketone body (Fujifilm, Cat# 415-7330, 411-73401 and 418-73891) levels were measured using colorimetric assays. Hepatic phosphatase activity was assessed with a phosphatase assay kit (G-Biosciences, Cat# 786-453). When required, samples were deproteinized using a trichloroacetic acid (TCA)-based deproteinization kit (Abcam, Cat# ab204708).

Western blots

Frozen liver tissues from mice were homogenized in RIPA lysis buffer (0.05 M tris-HCl pH 7.4, 0.15 M NaCl, 0.001 M EDTA, 1% triton X-100, 1% SDS, 1% IGEPAL, 1% sodium deoxycholate, 0.01 M NaF) supplemented with protease and phosphatase inhibitors (Roche). Protein extracts were separated on 10% SDS-PAGE (sodium dodecyl sulfate–polyacrylamide gel electrophoresis) and transferred to nitrocellulose membranes (Bio-Rad) using the Mini Trans-Blot® Cell system (Bio-Rad). Membranes were blocked in 10% non-fat milk dissolved in tris-buffered saline containing 0.1% tween 20 (TBST) and incubated overnight at 4°C with primary antibodies against HMGCS2 (Cell Signaling, Cat# 20940), ACAT1 (Invitrogen, Cat# PA5-19227), HMGCL (Invitrogen, Cat# PA5-21996), BDH1 (Novus Biologicals, Cat# NBP1-88673), phospho-AMPKα (Cell Signaling, Cat# 2535), AMPKα (Cell Signaling, Cat# 5831), phospho-CREB (Cell Signaling, Cat# 9198), CREB (Cell Signaling, Cat# 9197), ACADM (Abcam, Cat# ab92461), ACADL (Abcam, Cat# ab128566), and HSP90 (BD Biosciences, Cat# 610418). The following day, membranes were incubated for 1 hour at room temperature with horseradish peroxidase-conjugated secondary antibodies (anti-mouse IgG: Cell Signaling Cat# 7076, anti-rabbit IgG: Cell Signaling Cat# 7074, anti-goat IgG: Invitrogen Cat# A15963). Protein bands were then detected using an enhanced chemiluminescence (ECL) reagent (Thermo Fisher Scientific) and visualized on the ChemiDoc™ MP Imaging System (Bio-Rad), as previously described (2).

Quantitative real-time PCR

Total RNA was extracted from tissues using TRIzol™ Reagent (Thermo Fisher Scientific, Cat# 15596026) and reverse-transcribed into cDNA with the High-Capacity cDNA Reverse Transcription Kit (Thermo Fisher Scientific, Cat# 4368814), as previously described (3). Quantitative real-time PCR (RT-qPCR) was performed using SYBR Green Master Mix (Thermo Fisher Scientific) on a CFX Connect RT-PCR Detection System (Bio-Rad). The following primers were used: ***Lipe***, forward 5′-CCAGCCTGAGGGCTTACTG-3′ and reverse 5′-CTCCATTGACTGTGACATCTCG-3′; ***Plin1***, forward 5′-GGGACCTGTGAGTGCTTCC-3′ and reverse 5′-GTATTGAAGAGCCGGGATCTTTT-3′; ***Pnpla2***, forward 5′-GGATGGCGGCATTTCAGACA-3′ and reverse 5′-CAAAGGGTTGGGTTGGTTCAG-3′; ***Abhd5***, forward 5′-TGGTGTCCCACATCTACATCA-3′ and reverse 5′-

CAGCGTCCATATTCTGTTTCCA-3′; ***Gos2***, forward 5′-GTGAAGCTATACGTGCTGGG-3′ and reverse 5′-CCGTCTCAACTAGGCCGAG-3′; ***Hilpda***, forward 5′-TCGTGCAGGATCTAGCAGCAG-3′ and reverse 5′-GCCCAGCACATAGAGGTTCA-3′; ***Mgll***, forward 5′-CGGACTTCCAAGTTTTTGTCAGA-3′ and reverse 5′-GCAGCCACTAGGATGGAGATG-3′; ***Lipa***, forward 5′-AGTATTCACCGAATCCCTCG-3′ and reverse 5′-CTAGAATCTGCCAGCAAGCC-3′; ***Cpt1a***, forward 5′-GAGAAATACCCTGACTATGTG-3′ and reverse 5′-TGTGAGTCTGTCTCAGGGCTAG-3′; ***Cpt1b***, forward 5′-GCACACCAGGCAGTAGCTTT-3′ and reverse 5′-CAGGAGTTGATTCCAGACAGGTA-3′; ***Acox1***, forward 5′-GCCTGCTGTGTGGGTATGTCATT-3′ and reverse 5′-GTCATGGGCGGGTGCAT-3′; ***Acox2***, forward 5′-AACCCAGGGGATCGAGTGT-3′ and reverse 5′-CGCAGCTCAGTGTTTGGGAT-3′; ***Acads***, forward 5′-TGGCGACGGTTACACACTG-3′ and reverse 5′-GTAGGCCAGGTAATCCAAGCC-3′; ***Acadm***, forward 5′-GCTGGAGACATTGCCAATCA-3′ and reverse 5′-GGCGTCCCTCATCAGCTTCT-3′; ***Acadl***, forward 5′-TCTTTTCCTCGGAGCATGACA-3′ and reverse 5′-GACCTCTCTACTCACTTCTCCAG-3′; ***Cort***, forward 5′-GAACGGACATTTCAGTACCAGG-3′ and reverse 5′-CTTCATTTGCGAATGGTTTCACT-3′; ***Hadh***, forward 5′-TTGCCAGCAACACGTCTTCTT-3′ and reverse 5′-GAGGCCAGCAAATCGGTCTT-3′; ***Acaa2***, forward 5′-CTGCTACGAGGTGTGTTCATC-3′ and reverse 5′-AGCTCTGCATGACATTGCCC-3′; ***Pyc***, forward 5′-CTGAAGTTCCAAACAGTTCGAGG-3′ and reverse 5′-CGCACGAAACACTCGGATG-3′; ***cPepck***, forward 5′-CTGCATAACGGTCTGGACTTC-3′ and reverse 5′-CAGCAACTGCCCGTACTCC-3′; ***mPepck***, forward 5′-ATGGCTGCTATGTACCTCCC-3′ and reverse 5′-GCGCCACAAAGTCTCGAAC-3′; ***Fbpase***, forward 5′-CACCGCGATCAAAGCCATCT-3′ and reverse 5′-AGGTAGCGTAGGACGACTTCA-3′; ***G6pase***, forward 5′-CGACTCGCTATCTCCAAGTGA-3′ and reverse 5′-GTTGAACCAGTCTCCGACCA-3′; ***Gyk***, forward 5′-TGAACCTGAGGATTTGTCAGC-3′ and reverse 5′-CCATGTGGAGTAACGGATTTCG-3′; ***Pygl***, forward 5′-GAGAAGCGACGGCAGATCAG-3′ and reverse 5′-CTTGACCAGAGTGAAGTGCAG-3′; and ***Ppia***, forward 5′-GAGCTGTTTGCAGACAAAGTTC-3′ and reverse 5′-CCCTGGCACATGAATCCTGG-3′.

Drug affinity responsive target stability (DARTs) assay

To ascertain whether there is binding affinity between EMPA and HMGCS2, we performed a drug affinity responsive target stability (DARTs) assay ex vivo using liver protein lysates from C57BL/6J mice, incubated with or without EMPA. The principle of the DARTs assay rests on the observation that proteins become less susceptible to proteases when they bind with small molecules (4). Fresh liver tissues from 12-hour-fasted C57BL/6J mice were lysed using M-PER (Thermo Fisher Scientific) supplemented with protease and phosphatase inhibitors (Roche). Liver homogenates were centrifuged at 13,000 rpm for 10 minutes at 4 °C to remove cellular debris, and the supernatant was collected for the DARTs assay.

Protein concentration was quantified using the Bradford assay (Bio-Rad), and equal amounts of protein were proteolyzed in TNC buffer (50 mM tris-HCl, pH 8.0; 50 mM NaCl; 10 mM $CaCl_2$) to maintain consistent conditions that stabilize proteins and enable reliable protease activity. All steps were performed on ice or at 4 °C to help prevent premature protein degradation, as previously described (4). Liver protein lysates were incubated with either vehicle control (DMSO) or 3 mM EMPA for 30 minutes on ice, followed by an additional 30 minutes at room temperature. Digestion was performed using Pronase (Roche) at room temperature for 20 minutes and stopped by adding 2x SDS loading buffer, followed by immediate heating at 95 °C for 5 minutes. Samples were then subjected to SDS-PAGE, and Western blotting was carried out as described in the previous section, using antibodies against ACAT1 (Thermo Fisher Scientific, Cat# PA5-19227), HMGCS2 (Cell Signaling, Cat# 20940), HMGCL (Thermo Fisher Scientific, Cat# PA5-21996), and BDH1 (Novus Biologicals, Cat# NBP1-88673). HSP90 was used as a loading control to normalize signal intensity across all lanes. Protein bands were visualized using enhanced chemiluminescence (ECL) on a ChemiDoc™ MP Imaging System and quantified with ImageJ (version 1.53v). In this assay, increased band intensity was interpreted as enhanced EMPA-mediated protection of the protein from proteolytic degradation, indicating reduced protease-mediated cleavage and greater protein stability.

In vivo HMGCS2 activity

Liver protein lysates from 6-hour-fasted wildtype or knockout mice, treated with either vehicle control or EMPA, were used to quantify HMGCS2 enzymatic activity as previously described (5). Briefly, HMGCS2 activity was measured by assessing the conversion of acetyl-CoA and acetoacetyl-CoA to 3-hydroxy-3-methylglutaryl-CoA. The reaction mixture included 67 mM Tris-Cl (pH 8.0), 130 μM DTNB, 130 μM acetyl-CoA, and 7 μM acetoacetyl-CoA. The reaction was initiated by adding 200 μg of mouse liver protein lysate, and the formation of 3-hydroxy-3-methylglutaryl-CoA was monitored by detecting CoASH via dithiobisnitrobenzoic acid (DTNB). Absorbance at 412 nm was recorded every 30 seconds for 10 minutes. HMGCS2 activity unit is defined as the conversion of 1 μmol of substrate to product CoASH per minute.

In vitro measurement of HMGCS2 activity in human hepatocytes and HEK-293 cells

To evaluate the effect of EMPA on human HMGCS2 activity, primary human hepatocytes suspension cells, derived from normal healthy liver tissues, were obtained from ATCC (HepatoXcell™ Eco, Cat# PCS-450-012). Cells were cultured in 6-well plates at a density of 10^6 cells per well and maintained in HepatoXcell™ Maintenance Medium (Cat# PCS-450-034) according to the manufacturer's instructions. Cell viability was confirmed using the trypan blue exclusion assay. On the day of treatment, cells were

incubated in serum-free medium for 60 minutes and subsequently treated with either vehicle control or EMPA (100 μM) for 30 minutes. Following treatment, cells were collected by centrifugation and lysed in RIPA buffer supplemented with protease and phosphatase inhibitors. HMGCS2 enzymatic activity was then quantified from the cell lysates as described above. A similar experimental approach was used to measure HMGCS2 activity in HEK-293 cells (ATCC, Cat# CRL-11268), which were serum-starved for 60 minutes and subsequently treated with vehicle control or EMPA (100 μM) for 5 minutes.

In vitro kinetics assessment of HMGCS2

Recombinant human HMGCS2 protein, obtained from BioMart Inc. (Cat# HMGCS2-134H), was used to determine the median effective concentration (EC_{50}) of three SGLT2 inhibitors: EMPA, dapagliflozin (DAPA; MedChemExpress, Cat# HY-10450), and ertugliflozin (ERTU; MedChemExpress, Cat# HY-15461). The activity of 1 μg of recombinant HMGCS2 was measured as described above in the presence of increasing concentrations of each SGLT2 inhibitor (0.1–1000 μM). For kinetic analysis, HMGCS2 activity was assessed in reaction mixtures containing 1 μg of recombinant HMGCS2 and a fixed concentration of each SGLT2 inhibitor (100 μM), with acetyl-CoA concentrations ranging from 20 to 2000 μM as the substrate. Absorbance at 412 nm was recorded at room temperature over a 4-minute period. The data were fitted to the Michaelis-Menten equation using nonlinear least squares regression, with 95% confidence intervals, to determine the V_{max} and K_m values. A Lineweaver-Burk plot of the Michaelis-Menten data was used to identify the mode of HMGCS2 activation by each SGLT2 inhibitor, with linear regression fitting shown alongside 95% confidence intervals.

HMGCS2 mutagenesis

Two HMGCS2 mutants targeting threonine 91 (T91) were generated by BioMart Inc. In the first mutant, T91 was replaced with alanine (T91A) to mimic the dephosphorylated state, whereas in the second mutant, T91 was replaced with aspartic acid (T91D) to mimic the phosphorylated state. Briefly, human HMGCS2 (UniProt: P54868) constructs carrying the T91A and T91D substitutions were produced by custom gene synthesis. The codon-optimized genes were cloned into the pET-28a(+) vector under the control of a T7 promoter, incorporating an N-terminal His-tag. Sequence verification was performed by Sanger sequencing using the universal primers T7 (5′-TAATACGACTCACTATAGGG-3′) and T7Ter (5′-TGCTAGTTATTGCTCAGCGG-3′). Sequencing confirmed the expected substitutions (T91A: ACG → GCG; T91D: ACG → GAT) and verified the integrity of the full-length open reading frame prior to E. coli transformation for recombinant protein expression. Subsequently, the enzymatic activity of 1 μg of wild-type or mutant HMGCS2 was measured as described above in the presence of increasing concentrations of EMPA.

Molecular docking and binding simulation

Molecular docking was performed using PyRx (version 0.8) (6) to predict the binding affinity between SGLT2 inhibitors and HMGCS2. The crystal structure of HMGCS2 (PDB ID: 2WYA) was obtained from the Protein Data Bank. Water molecules and heteroatoms were removed, and the protein was energy minimized using DeepView/Swiss-PdbViewer (version 4.1.0). Ligand structures were retrieved from PubChem and DrugBank, and their geometries were optimized using Open Babel integrated in PyRx. A grid box of 60 × 60 × 60 points was centered around the enzyme's active site with a grid spacing of 0.5 Å and an exhaustiveness of 8. Docking simulations were conducted using AutoDock Vina in PyRx, with ten independent docking runs for each ligand. The resulting ligand-protein complexes were ranked based on binding affinity (kcal/mol). PyMOL (version 3.1.3) and Discovery Studio Visualizer (version v24.1.0) was used to visualize the docked structure of protein and ligand complexes.

Molecular dynamics simulations

Molecular dynamics (MD) simulations were conducted using GROMACS (version 2023.3) (7) to assess the stability and dynamic behavior of the protein-ligand complex. The CHARMM27 all-atom force field was used for protein parametrization, while ligand topology was generated using the SwissParam platform (8). The system was solvated in a cubic box of TIP3P water model with a minimum distance of 1.0 nm from the box edges, and neutralized by adding counterions (Na^+ or Cl^-). Energy minimization was performed using the steepest descent algorithm to eliminate steric clashes. The system was equilibrated in two phases: an NVT ensemble for 100 ps to stabilize the temperature, followed by an NPT ensemble for 100 ps to stabilize the pressure. The final MD simulation was run for 100 ns with a time step of 2 fs under periodic boundary conditions. Temperature and pressure were maintained at 300 K and 1 bar using the V-rescale thermostat and Parrinello-Rahman barostat, respectively. Trajectory fitting, as well as rotational and translational adjustments, were performed to facilitate smoother visualization of the MD results.

HMGCS2 immunoprecipitation

Frozen liver tissue samples collected from 6-hour fasted mice treated with EMPA or VC underwent homogenization and solubilization in IP buffer containing protease and phosphatase inhibitor, using a TissueLyser for 1 hour. For each sample, 1.7 mg of protein was incubated with 6 µl of HMGCS2 antibody overnight at 4°C with rotation. The antibody-containing samples were then incubated with 50 µl of Pierce Protein A/G Magnetic Agarose Beads (Thermo Fisher Scientific) previously washed three times in 0.1% bovine serum albumin. Incubation with the beads proceeded for 3 hours at 4°C with continuous rotation,

followed by ten washes with phosphate buffered-saline (PBS) to remove unbound proteins. After the washes, reduction and alkylation of the beads were performed with 5 mM tris(2-carboxyethyl)phosphine (Sigma) and 20 mM chloroacetamide (Sigma). Samples were incubated for 1 hour at room temperature with shaking (vortexing), and then trypsin digestion was initiated by adding sequencing-grade trypsin at a 1:50 enzyme-to-substrate ratio (1 mg trypsin: 50 mg protein) and incubating overnight at 37°C with shaking (500 rpm). The following day, samples were desalted using C18 microcolumns, and the eluate was dried in a speed vacuum.

Phosphoproteomics

The digested peptides resulted from IP underwent solubilization in 5% acetonitrile-4% formic acid. A Pepmap Neo C18 pre-column (Thermo Fisher Scientific) was used for sample loading. Peptide separation occurred on a 25 cm Aurora Column (IonOpticks, 75-µm i.d. by 250 mm) with a 56-minute gradient of 10% to 30% acetonitrile-0.2% formic acid at a 300-nl/min flow rate. A Neo Vanquish UHPLC, coupled to an Orbitrap Ascend Tribrid Mass Spectrometer (Thermo Fisher Scientific, San Jose, CA), facilitated the separation and analysis. Full MS spectra were acquired at a resolution of 120,000 with a 50 ms injection time. These spectra were followed by tandem MS (MS/MS) acquisition on the most abundant multiply charged precursor ions for 3 seconds. Tandem MS experiments employed higher-energy collision dissociation (HCD) at a 27% collision energy, 50% AGC, 22,500 resolution, and a 43 ms injection time. Data processing was performed using PEAKS X Pro (Bioinformatics Solutions, Waterloo, ON) and a Uniprot human database (20,366 entries). Mass tolerances for precursor and fragment ions were 10 ppm and 0.01 Da, respectively. Carbamidomethyl (C) was a fixed modification. Variable post-translational modifications selected were oxidation (M), deamidation (NQ), phosphorylation (STY), and acetylation (N-term).

Statistical analysis

Sample sizes were determined based on prior experience with each assay to ensure adequate statistical power for detecting biologically meaningful differences. Statistical analyses were conducted using either unpaired Student's t-tests or one-way ANOVA followed by Fisher's LSD post-hoc test, depending on the experimental design, as indicated in the figure legends. All analyses were performed using GraphPad Prism software 10, with statistical significance set at $P < 0.05$. Data are expressed as means ± standard error of the mean (SEM), unless otherwise noted in the figure legends.

Study approval

All animal experiments were reviewed and approved by the Animal Care Committee of the Université de Montréal (Montréal, Québec, Canada; Protocol #23-041). All procedures were conducted in accordance with the guidelines of the Canadian Council on Animal Care (CCAC).

Data availability

All data supporting the findings of this study are included within the article and its supplementary materials. Mass spectrometry (MS) raw data are available from the corresponding author upon reasonable request (rami.al.batran@umontreal.ca).

Acknowledgements

This work was supported by a Project Grant (PJT-195730) from the Canadian Institutes of Health Research (CIHR) to R.A. R.A. is a Research Scholar of the Fonds de recherche du Québec – Santé (FRQS) and a New Investigator in the Kidney Research Scientist Core Education and National Training (KRESCENT) Program and the Heart and Stroke Foundation of Canada (HSF). Proteomics analyses were performed at the Centre for Advanced Proteomics Analyses (CAPA), a node of the Canadian Genomics Innovation Network, which is supported by the Government of Canada through Genome Canada.

References:

1. Mourad S, et al. A high-fat diet supplemented with medium-chain triglycerides ameliorates hepatic steatosis by reducing ceramide and diacylglycerol accumulation in mice. *Exp Physiol.* 2024;109(3):350-64.
2. Mechchate H, et al. Defective muscle ketone body oxidation disrupts BCAA catabolism by altering mitochondrial branched-chain aminotransferase. *Am J Physiol Endocrinol Metab.* 2023;324(5):E425-e36.
3. Gopal K, et al. Loss of muscle PDH induces lactic acidosis and adaptive anaplerotic compensation via pyruvate-alanine cycling and glutaminolysis. *J Biol Chem.* 2023;299(12):105375.
4. Lomenick B, et al. Target identification using drug affinity responsive target stability (DARTS). *Proc Natl Acad Sci U S A.* 2009;106(51):21984-9.
5. Andrew Skaff D, and Miziorko HM. A visible wavelength spectrophotometric assay suitable for high-throughput screening of 3-hydroxy-3-methylglutaryl-CoA synthase. *Anal Biochem.* 2010;396(1):96-102.
6. Dallakyan S, and Olson AJ. Small-molecule library screening by docking with PyRx. *Methods Mol Biol.* 2015;1263:243-50.
7. Lemkul J. From proteins to perturbed Hamiltonians: a suite of tutorials for the GROMACS-2018 molecular simulation package [article v1. 0], living J. *Comput Mol Sci.* 2019;1(10.33011).
8. Zoete V, et al. SwissParam: a fast force field generation tool for small organic molecules. *J Comput Chem.* 2011;32(11):2359-68.
