# Supplementary material for: SGLT2 inhibitors enhance ketogenesis by acting as allosteric activators of the mitochondrial enzyme HMGCS2
Source: J Clin Invest. 2026 Jul 1;136(13):e192333. doi: 10.1172/JCI192333 (PMC13318103; doi:10.1172/JCI192333)
Supplement: Unedited blot and gel images [file jci-136-192333-s234.pdf]

Unmodified and unprocessed immunoblotting images related to Fig. 1J

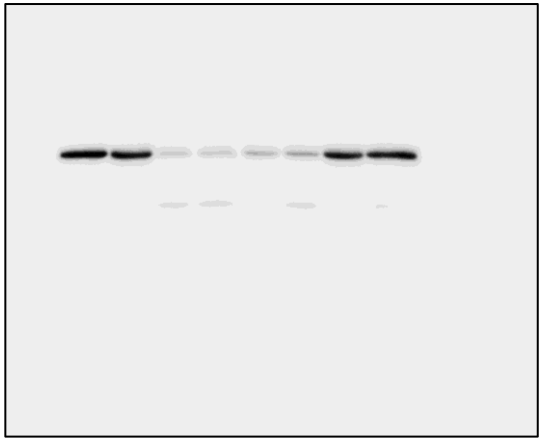

ACAT1

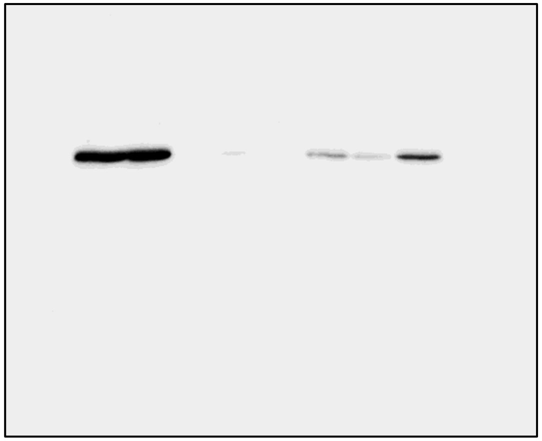

HMGCS2

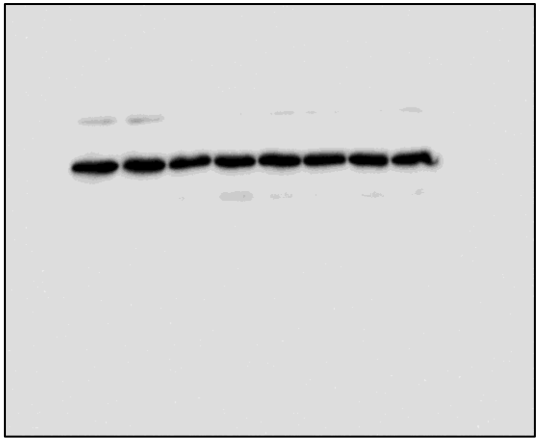

HMGCL

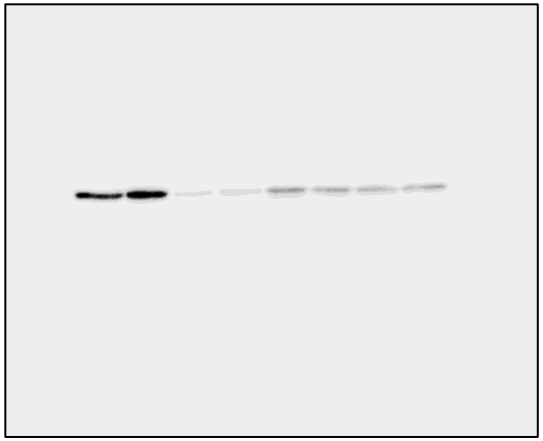

BDH1

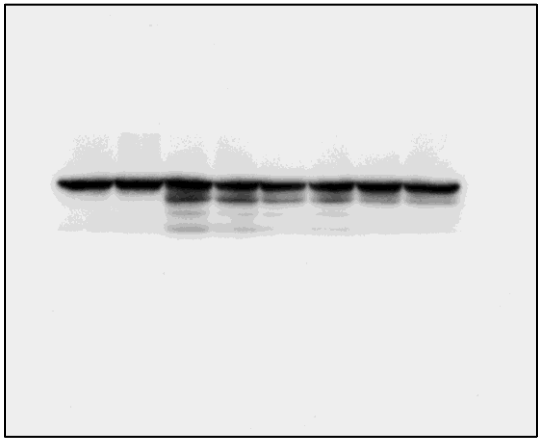

HSP90

Unmodified and unprocessed immunoblotting images related to Supp Fig. 3A

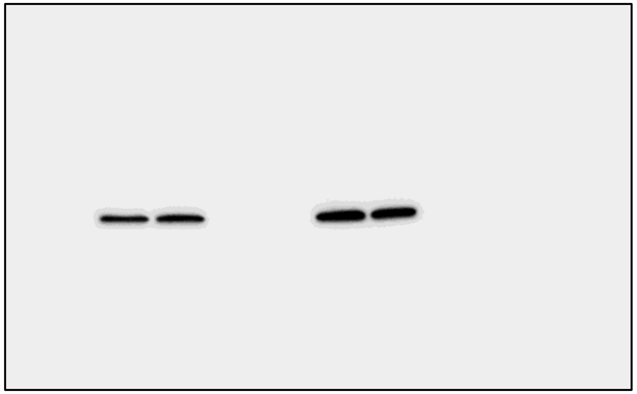

HMGCS2 (Liver)

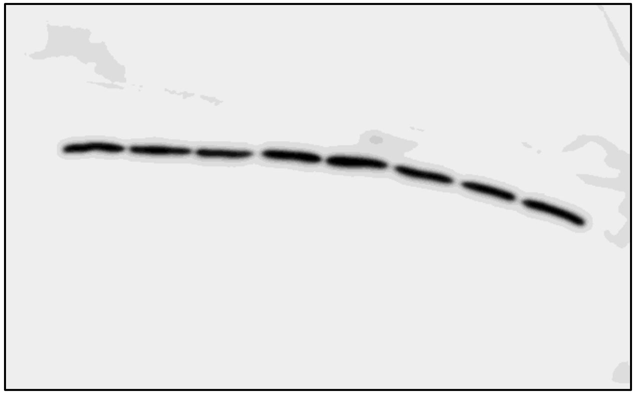

HMGCS2 (Kidney)

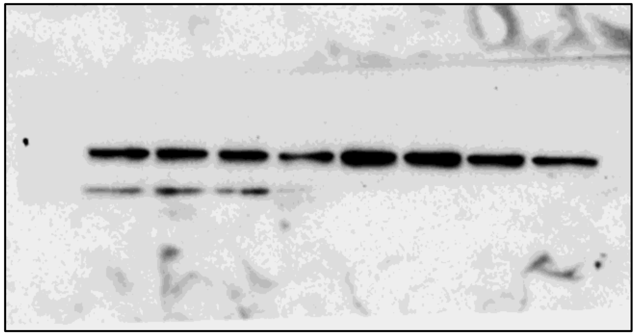

HSP90 (Liver)

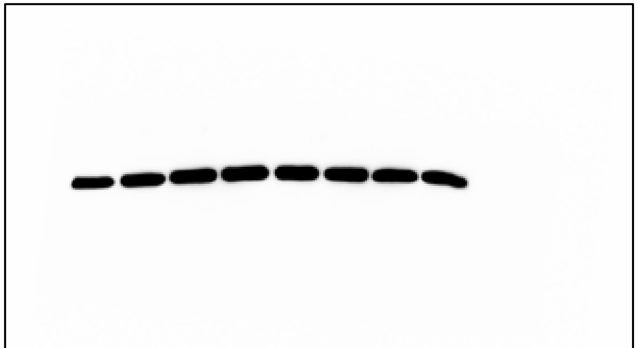

HSP90 (Kidney)

Unmodified and unprocessed immunoblotting images related to Supp Fig. 3B

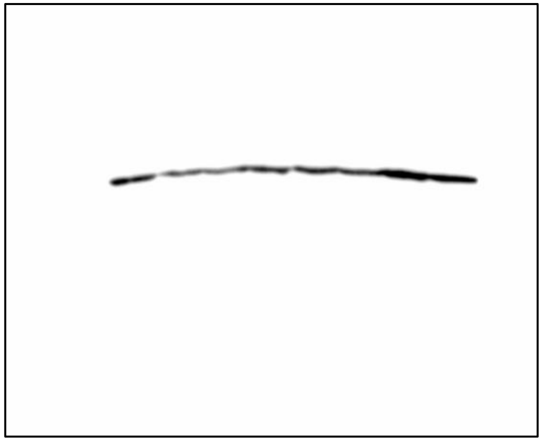

ACAT1

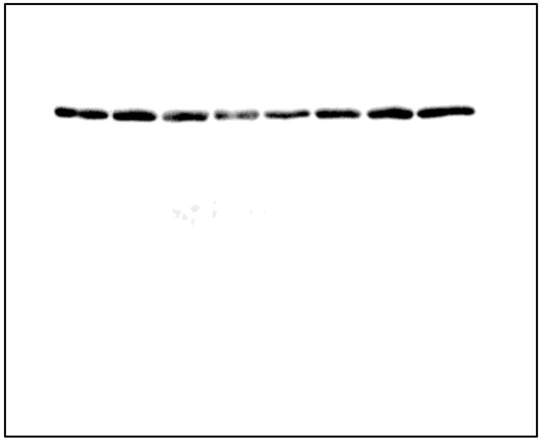

HMGCL

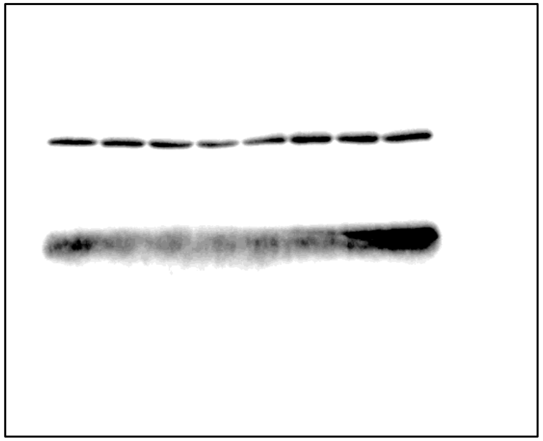

BDH1

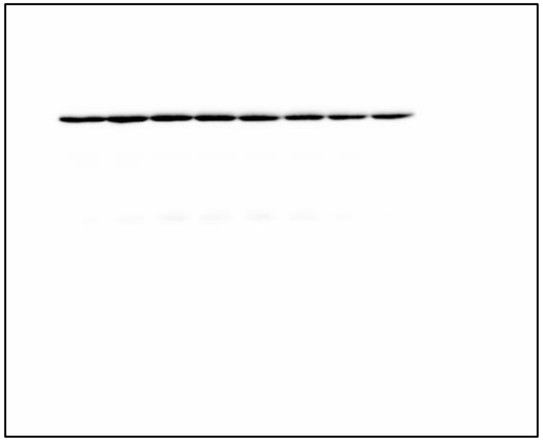

HSP90

Unmodified and unprocessed immunoblotting images related to Supp Fig. 3N

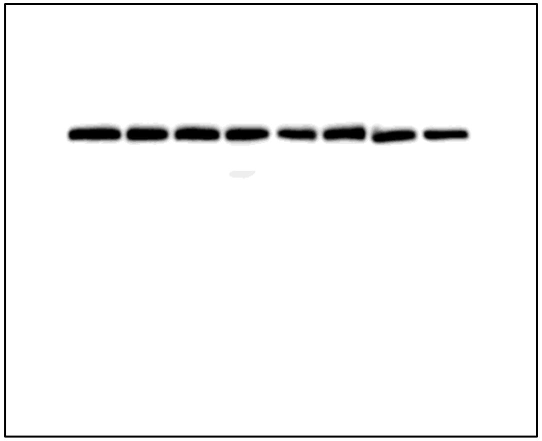

P-AMPKα

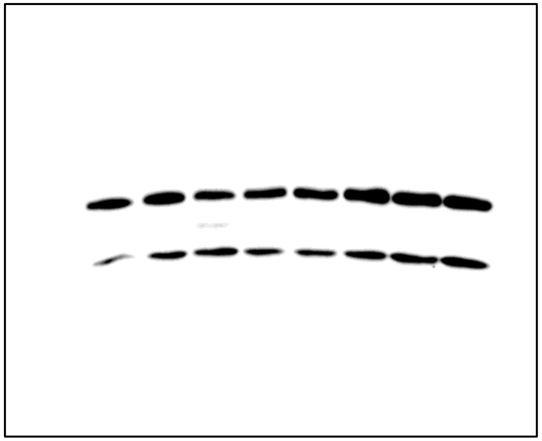

AMPKα

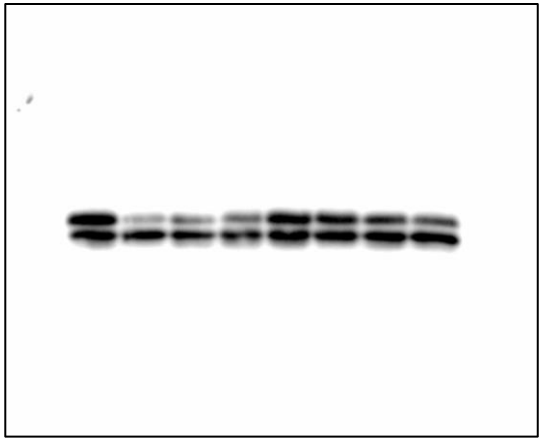

P-CREB

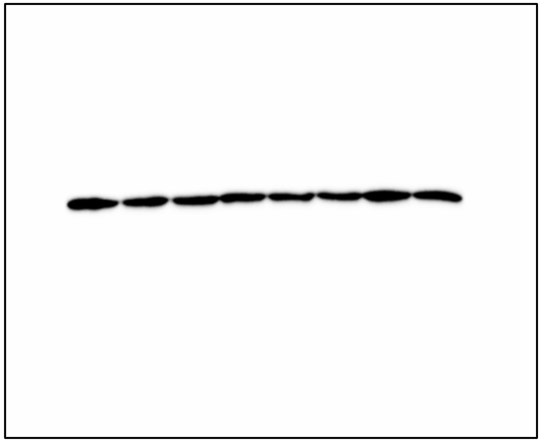

CREB

Unmodified and unprocessed immunoblotting images related to Supp Fig. 3O

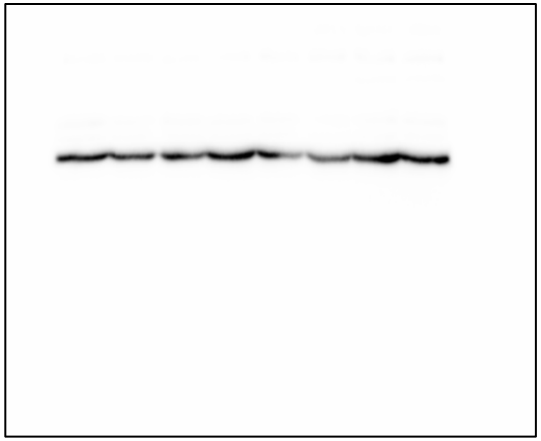

ACADM

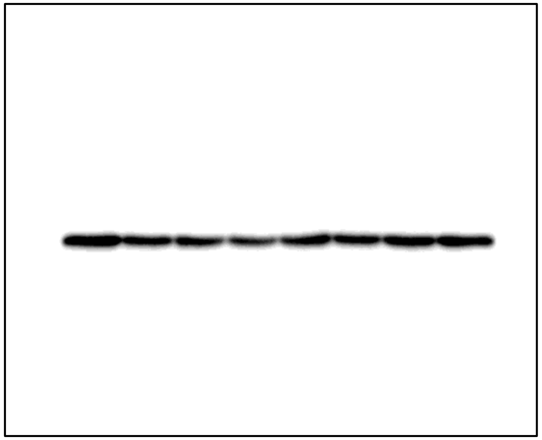

ACADL

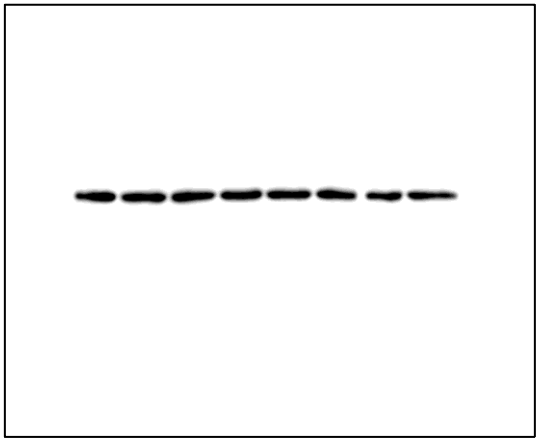

HSP90
